# MicroRNAs as the critical regulators of cell migration and invasion in thyroid cancer

**DOI:** 10.1186/s40364-022-00382-4

**Published:** 2022-06-04

**Authors:** Amir Abbas Hamidi, Negin Taghehchian, Zahra Basirat, Amir Sadra Zangouei, Meysam Moghbeli

**Affiliations:** 1 Student Research Committee, Faculty of Medicine, Mashhad University of Medical Sciences, Mashhad, Iran; 2 Medical Genetics Research Center, Mashhad University of Medical Sciences, Mashhad, Iran; 3 Department of Medical Genetics and Molecular Medicine, School of Medicine, Mashhad University of Medical Sciences, Mashhad, Iran

**Keywords:** Thyroid cancer, miRNA, Invasion, Metastasis, Migration, Panel marker

## Abstract

Thyroid cancer (TC) is one of the most frequent endocrine malignancies that is more common among females. Tumor recurrence is one of the most important clinical manifestations in differentiated TC which is associated with different factors including age, tumor size, and histological features. Various molecular processes such as genetic or epigenetic modifications and non-coding RNAs are also involved in TC progression and metastasis. The epithelial-to-mesenchymal transition (EMT) is an important biological process during tumor invasion and migration that affects the initiation and transformation of early-stage tumors into invasive malignancies. A combination of transcription factors, growth factors, signaling pathways, and epigenetic regulations affect the thyroid cell migration and EMT process. MicroRNAs (miRNAs) are important molecular factors involved in tumor metastasis by regulation of EMT-activating signaling pathways. Various miRNAs are involved in the signaling pathways associated with TC metastasis which can be used as diagnostic and therapeutic biomarkers. Since, the miRNAs are sensitive, specific, and non-invasive, they can be suggested as efficient and optimal biomarkers of tumor invasion and metastasis. In the present review, we have summarized all of the miRNAs which have been significantly involved in thyroid tumor cells migration and invasion. We also categorized all of the reported miRNAs based on their cellular processes to clarify the molecular role of miRNAs during thyroid tumor cell migration and invasion. This review paves the way of introducing a non-invasive diagnostic and prognostic panel of miRNAs in aggressive and metastatic TC patients.

## Background

Thyroid cancer (TC) is the most common head and neck cancer that involves 91.5% of all endocrine malignancies. It is the 7th common cause of cancer in females and the 14th among male. Based on the American Cancer Association studies, most of the new TC cases have been diagnosed in females under the age of 65 [1]. The major cause of difference between females and males incidence rates can be related to the estrogen receptors genes polymorphisms in females which can considerably increase the tumor cell proliferation [1, 2].

TC is histopathologically categorized into different subtypes including well-differentiated TC (WDTC), poorly-differentiated TC (PDTC), and undifferentiated anaplastic thyroid carcinomas (ATC). WDTC also contains two subtypes: papillary TC (PTC) and follicular TC (FTC) (Table 1). PTCs are the most common differentiated thyroid carcinomas (80–85% of all thyroid malignancies) [3], while the ATC is the least common and most aggressive type with poor prognosis. FTC is also the 2nd most frequent malignancy originating from follicular cells which is categorized into minimally invasive FTC (MI-FTC) and widely invasive FTC (WI-FTC) [4]. Most of differentiated TC with slow clinical progression causes distant metastasis and reduced survival rate. Tumor recurrence is one of the most important clinical manifestations in differentiated TCs. Different risk factors including age, tumor size, histological features, and metastasis influence the recurrence of differentiated TC [5–7].

**Table 1:** Histopathological classification of thyroid cancer

Thyroid cancer
➣ Follicular origin
● Undifferentiated
✦ Anaplastic thyroid cancer
● Differentiated
▪ Well differentiated
✦ Papillary thyroid cancer
✦ Follicular thyroid cancer
✦ Hurthle cell cancer
▪ Poorly differentiated
➣ Para follicular origin
✦ Medullary thyroid cancer

Environmental factors, lifestyles, or a combination of these factors are supposed to be possible causes for the raising trend of TC incidence [8]. Gender, weight, diabetes mellitus, ionizing radiation, pregnancy, genetic factors, thyroid disorders, hormones, and dietary pattern are among the risk factors of TC [9–11]. Various molecular processes such as genetic or epigenetic modifications and non-coding RNAs are involved in TC progression and metastasis [3]. The epithelial-to-mesenchymal transition (EMT) is an important biological process during tumor invasion and migration in which the epithelial cells can lose their cell-cell adhesion and turn into mesenchymal phenotype. It affects the initiation and transformation of early-stage tumors into invasive malignancies [12]. EMT is triggered by the down regulation of epithelial markers like CDH1 and up regulation of mesenchymal markers like vimentin and CDH2 [13]. EMT can be the basic cause of tumor invasion and migration [14]. A combination of transcription factors, growth factors, signaling cascades, epigenetic regulations and tumor environment affect the thyroid cell migration and EMT process [14]. There is a correlation between TC poor prognosis and genetic alterations in EMT-related genes and transcription factors. EMT-related factors suppress the expression of E-cadherin (E-CAD) and are related to tumor progression, local invasion, and metastasis [15, 16]. Molecular mechanisms of EMT and cell migration are regulated by WNT, TGFβ, MAPK, and phosphatidylinositol-3-kinase (PI3K) pathways [14, 17]. Some of the essential transcription factors such as Twist, Snail1-2, and ZEB1-2 are the main triggers of the EMT process [18–21]. WNT, NOTCH, and TGF-β are also the major signaling pathways to activate the EMT-promoting transcription factors [22, 23]. Snail-2 is positively regulated by NOTCH signaling that results in EMT induction following the CDH1 inhibition [24].

MicroRNAs (miRNAs) are important molecular factors involved in tumor metastasis by regulation of EMT-activating signaling pathways [25]. Specific patterns of miRNA expression have been identified in thyroid carcinomas [26–28]. MiRNA profiling is widely being introduced as biomarkers in a variety of diseases which can be used as prognostic and predictive factors in the clinical research [29]. Various studies have demonstrated that miRNAs deregulation plays a pivotal role in tumor cell invasion and migration [30, 31]. Different miRNAs have been participated in the signaling pathways of thyroid tumor metastasis and migration which could be applied as novel biomarkers for treatment and early stage diagnosis [32]. Since, the miRNAs are sensitive, specific, and non-invasive; they can be suggested as efficient and optimal biomarkers of tumor invasion and metastasis. The miRNAs expression can be recognized in body fluids and tissues which are associated with developmental stages, tissues, and external stimulation. Circulating miRNAs in body fluids are frequently used as non-invasive tumor biomarkers [33, 34]. There are different miRNA quantification methods including sequencing, hybridization, and amplification based methods. NGS, microarray, and real-time PCR have been widely employed to discover circulating miRNAs [35]. Bead-based flowcytometry assays were also developed to solve the cross-hybridization challenge of miRNAs in the microarray procedures [33, 36]. Some other amplification methods such as rolling circle amplification (RCA), strand displacement amplification (SDA), and loop-mediated isothermal amplification (LAMP) have been also employed to assess the miRNAs functions [37]. Liquid biopsy has minimal invasion for the assessment of tumor profile in comparison with tumor biopsy. However, this method has some limitations including short half-life of circulating miRNAs, low concentration, and contamination with normal cells [38–40]. In present review, we have summarized all of the miRNAs that have been involved in thyroid tumor cells migration and invasion to pave the way of introducing a non-invasive diagnostic and prognostic panel of miRNAs in TC. We have categorized all of the reported miRNAs based on their cellular processes to clarify the molecular role of miRNAs during thyroid tumor cell migration and invasion (Table 2).

**Table 2:** All of the microRNAs associated with tumor cell migration and invasion in thyroid cancer

**microRNA**	**Target**	**Results**	**Clinical Application**	**Samples**	**Country**	**Year**	**Study**
PI3K/AKT Signaling							
miR-497	AKT3	MiR-497 inhibited PTC cells migration and invasion through AKT3 suppression.	Diagnosis	43 patients 5 cell lines	China	2017	Zhuang [41]
miR-145	AKT3	MiR-145 targeted the AKT3, N-cadherin, and HIF1a during TC progression.	Diagnosis and prognosis (liquid biopsy)	3 cell lines	USA	2014	Boufraqech [42]
miR-217	AKT3	The miR-217 down regulation was also associated with clinical stage and lymphatic metastasis.	Diagnosis and prognosis	58 patients 4 cell lines	China	2017	Lin [43]
miR-203	AKT3	MiR-203 up regulation and AKT3 down regulation reduced tumor cell migration.	Diagnosis	105 patients 6 cell lines	China	2020	You [44]
miR-338-3p	AKT3	MiR-338-3p significantly suppressed the cell migration of TC cells by targeting AKT3.	Diagnosis and prognosis	48 patients 4 cell lines	China	2017	Sui [45]
miR-29a	AKT3	MiR-29a suppressed PTC cell migration and invasion by targeting AKT3.	Diagnosis and prognosis	30 patients 1 cell line	China	2016	Li [46]
Let-7a	AKT2	Let-7a significantly inhibited the PTC migration and invasion by targeting AKT2.	Diagnosis	47 patients 3 cell lines	China	2017	Zhou [47]
miR-718	PDPK1	MiR-718 suppressed PTC cell migration through PDPK1 targeting.	Diagnosis	15 patients 2 cell lines	China	2018	Wang [48]
miR-125b	PIK3CD	MiR-125b targets PIK3CD and inhibits the ATC cell migration.	Diagnosis	30 patients 3 cell lines	China	2017	Bu [49]
miR-363-3p	PIK3CA	MiR-363-3p repressed migratory and invasive behaviors of PTC cells via PIK3CA targeting.	Diagnosis and prognosis	30 patients 4 cell lines	China	2017	Liu [50]
miR-135a-5p	PPM1E	LINC01087 induced TC cell migration via miR-135a-5p/PPM1E axis.	Diagnosis	30 patients 2 cell lines	China	2022	Yin [51]
miR-646	HNRNPA1	CircTIAM1 induced PTC cell migration through the miR-646/HNRNPA1 axis	Diagnosis	60 patients 6 cell lines	China	2022	Zhang [52]
MiR-30b-5p	GALNT7	MiR-30b-5p inhibited PTC cell proliferation and migration by GALNT7 targeting	Diagnosis and prognosis	60 patients 3 cell lines	China	2021	Wang [53]
miR-141	IRS2	MiR-141 inhibited cell migration and invasive capability of thyroid tumor cells by IRS2 targeting.	Diagnosis and prognosis	30 patients 1 cell line	China	2016	Dong [54]
miR-766	IRS2	MiR-766 inhibited the malignant activity of PTC cells through IRS2 suppression.	Diagnosis and prognosis	47 patients 4 cell lines	China	2019	Zhao [55]
miR-215	ARFGEF1	MiR-215 suppressed PTC metastasis by regulating the AKT/GSK-3β/Snail cascade and ARFGEF1 targeting.	Diagnosis and prognosis	48 patients 4 cell lines	China	2019	Han [56]
miR-451a	MIF	MiR-451a down regulation in PTC samples was significantly correlated with the aggressive PTC and advanced TNM stage.	Diagnosis and prognosis	19 patients 5 cell lines	Italy	2016	Minna [57]
miR-146b	PTEN	MiR-146b is involved in regulation of PTEN/PI3K/AKT pathway during thyroid tumor aggressiveness.	Diagnosis	2 cell lines	Spain	2018	Ramírez-Moya [58]
MiR-671-5p	TRIM14	MiR-671-5p inhibited the PTC cell migration by TRIM14 targeting.	Diagnosis	2 cell lines	China	2021	Wang [59]
miR-215-5p	TRIM44	CircWDR27 increased PTC cell migration by regulation of the miR-215-5p/TRIM44 axis.	Diagnosis	42 patients 2 cell lines	China	2021	Wang [60]
miR-627	TRIM44	LINC00958 increased PTC cell migration by miR-627 sponging and TRIM44 up regulation.	Diagnosis	10 patients 3 cell lines	China	2022	Li [61]
WNT and Hippo Signaling							
miR-3619-3p	b-catenin	MiR-3619-3p promoted the migratory and invasive capability of PTC cells through increased b-catenin mRNA stability.	Diagnosis and prognosis	36 patients 4 cell lines	China	2019	Yu [62]
miR-126	LRP6	MiR-126 up regulation suppressed PTC cells migration and in vivo tumor growth through LRP6 suppression.	Diagnosis and prognosis	30 patients 2 cell lines	China	2015	Wen [63]
miR-381-3p	LRP6	MiR-381-3p was associated with PTC metastasis through LRP6 down regulation.	Diagnosis and prognosis	8 patients 4 cell lines	China	2018	Kong [64]
miR-26a-5p	WNT5a	MiR-26a-5p inhibited the PTC cell growth, invasion, and metastasis via WNT5a suppression.	Diagnosis	58 patients 3 cell lines	China	2019	Shi [65]
miR-329	WNT1	MiR-329 inhibited the cell migration, invasion, and in vivo tumor growth via WNT1 suppression.	Diagnosis	20 patients 3 cell lines	China	2018	Wu [66]
miR-574-5p	FOXN3	MiR-574-5p mediated TC cell proliferation and migration through the Wnt/β-catenin pathway by targeting FOXN3.	Diagnosis	2 cell lines	China	2020	Zhang [67]
miR-1270	SCAI	MiR-1270 was involved in PTC progression through SCAI targeting.	Diagnosis	16 patients 5 cell lines	China	2019	Yi [68]
miR-625-3p	AEG1	MiR-625-3p enhanced TC cell migration and invasion by AEG-1 up regulation and triggering WNT pathway.	Diagnosis	20 patients 2 cell lines	China	2018	Fang [69]
miR-564	AEG1	Negative role of miR-564 on PTC migration and invasion via AEG1 inhibition.	Diagnosis and prognosis	47 patients 4 cell lines	China	2019	Song [70]
miR-205	YAP1	MiR-205 inhibited the Hippo signaling by targeting YAP1 and prevented TC cell migration and invasion.	Diagnosis	132 patients 4 cell lines	China	2018	Li [71]
miR34c5p	CRABP2	LINC01816 increased TC cell migration and EMT process via miR34c5p/CRABP2 axis.	Diagnosis	10 patients 5 cell lines	China	2021	Zhao [72]
TGFβ Signaling							
miR-663	TGFβ	MiR-663 reduced PTC cell invasion, migration, and EMT via TGFβ inhibition.	Diagnosis and prognosis	91 patients 2 cell lines	China	2016	Wang [73]
miR-146b-5p	SMAD4	MiR-146b-5p was involved in PTC cell migration through SMAD4 targeting.	Diagnosis	2 cell lines	Brazil	2016	Lima [74]
miR-483	PARD3	MiR-483 up regulated the Tiam1-Rac1 signals through PARD3 down regulation.	Diagnosis	80 patients 4 cell lines	China	2019	Zhang [75]
Cytokines							
miR-155	SOCS1	MiR-155 enhanced tumor progression by SOCS1 suppression.	Diagnosis and prognosis	31 patients 2 cell lines	China	2019	Zhang [76]
miR-25	SOCS4	MiR-25 enhanced the TC migration via targeting SOCS4.	Diagnosis	35 patients 2 cell lines	China	2015	Mei [77]
miR-126	CXCR4	MiR-126 reduced tumor cell migration and invasion via CXCR4 targeting.	Diagnosis	20 patients 2 cell lines	China	2016	Qian [78]
miR-223-3p	CXCR4	DUXAP8 induced PTC cell migration and proliferation through the regulation of miR-223-3p/CXCR4 axis.	Diagnosis	4 cell lines	China	2021	Liu [79]
miR-137	CXCL12	MiR-137 inhibited PTC cell proliferation, migration, and invasion by targeting CXCL12.	Diagnosis and prognosis	30 patients 1 cell line	China	2016	Dong [80]
MiR-455-5p	CXCL12	MiR-455-5p suppressed EMT and invasion in MTC cells by CXCL12 targeting.	Diagnosis and prognosis	28 patients 2 cell lines	China	2021	Zheng [81]
miR-1	CXCR4 and SDF-1	MiR-1 regulated the TC cell migration through CXCR4 and SDF-1 suppression.	Diagnosis	6 cell lines	Italy	2011	Leone [82]
miR-873-5p	CXCL16	MiR-873-5p inhibited PTC cells migration and invasion by targeting CXCL16.	Diagnosis	30 patients 6 cell lines	China	2020	Wang [83]
Kinases							
miR-497	BDNF	MiR-497 suppressed cells migration through BDNF targeting.	Diagnosis and prognosis	48 patients 1 cell line	China	2017	Wang [84]
miR-26b-5p	MET	Circ-0079558 promoted the PTC cell proliferation and migration, while reduced apoptosis via miR-26b-5p/MET/AKT axis.	Diagnosis	30 patients 2 cell lines	China	2021	Zheng [85]
miR-375	ERBB2	MiR-375 reduced PTC cell migration and invasion through ERBB2 suppression.	Diagnosis	60 patients 3 cell lines	China	2016	Wang [86]
miR-195	VEGFA	CircPVT1 promoted the PTC cell migration by miR-195 sponging and subsequent VEGFA up regulation.	Diagnosis	50 patients 4 cell lines	China	2021	Zeng [87]
miR-520a-3p	JAK1	MiR-520a-3p inhibited the JAK-STAT signaling by JAK1 suppression which inhibited EMT and migration of PTC cells.	Diagnosis	137 patients 6 cell lines	China	2019	Bi [88]
miR-361-5p	ROCK1	MiR-361-5p inhibited PTC cells migration by ROCK1 targeting.	Diagnosis and prognosis	48 patients 4 cell lines	China	2018	Li [89]
miR-150-5p	ROCK1	MiR-150-5p inhibited PTC cell migration through ROCK1.	Diagnosis and prognosis	45 patients 5 cell lines	China	2017	Cheng [90]
miR-128	SPHK1	MiR-128 reduced TC cell migration and invasion via targeting SPHK1.	Diagnosis	30 patients 8 cell lines	China	2019	Cao [91]
miR-577	SPHK2	MiR-577 inhibited PTC cells migration and invasion through SPHK2 suppression.	Diagnosis	35 patients 4 cell lines	China	2017	Xue [92]
miR-613	SPHK2	MiR-613 inhibited the PTC cell migration, invasion, and in vivo growth by targeting SPHK2.	Diagnosis	20 patients 4 cell lines	China	2016	Qiu [93]
miR-539	CARMA1	MiR-539 is a modulator of TC cell migration and invasion by targeting CARMA1.	Diagnosis	31 patients 1 cell line	China	2015	Gu [94]
miR-675	MAPK1	MiR-675 inhibited PTC cell migration and in vivo growth through MAPK1 regulation.	Diagnosis and prognosis	57 patients 4 cell lines	China	2019	Wang [95]
miR-7	PAK1	MiR-7 inhibited TC cells migration and invasion through targeting PAK1.	Diagnosis and prognosis	32 patients 2 cell lines	China	2016	Yue [96]
miR-101	RAC1	MiR-101 repressed PTC cell invasion and migration through RAC1 targeting.	Diagnosis	16 patients 3 cell lines	China	2014	Wang [97]
Transcriptional Regulation And Transcription Factors							
let-7b	HMGA2	Let-7b inhibited PTC cell migration and invasion through HMGA2 targeting.	Diagnosis	20 patients 5 cell lines	China	2017	Li [98]
let-7e	HMGB1	Let-7e suppressed PTC migration through HMGB1 targeting.	Diagnosis	2 cell lines	China	2019	Ding [99]
miR-212	SIRT1	MiR-212 inhibited cell proliferation, migration, and in vivo growth through SIRT1 targeting.	Diagnosis and prognosis	42 patients 4 cell lines	China	2018	Li [100]
miR-144	ZEB1 and ZEB2	MiR-144 suppressed migration of thyroid tumor cells through targeting ZEB1 and ZEB2.	Diagnosis	42 patients 2 cell lines	China	2015	Guan [101]
miR-429	ZEB1	MiR-429 suppressed TC cell migration through ZEB1 targeting.	Diagnosis	59 patients 3 cell lines	China	2019	Wu [102]
miR-335	ZEB2	MiR-335 suppressed TC cell migration through ZEB2 targeting.	Diagnosis	59 patients 5 cell lines	China	2017	Kan [103]
miR-194	ZEB1	CircVANGL1 promoted PTC cell migration and EMT process miR-194 sponging and ZEB1 up regulation.	Diagnosis and prognosis	77 patients 3 cell lines	China	2022	Xiang [104]
miR-30a	E2F7	MiR-30a inhibited the PTC cell migration by targeting E2F7.	Diagnosis	15 patients 2 cell lines	China	2019	Guo [105]
miR-544	YY1	MiR-544 suppressed tumor metastasis by targeting YY1.	Diagnosis	40 patients 4 cell lines	China	2019	Wang [106]
miR-211-5p	SOX11	MiR-211-5p suppressed TC cells migration and invasion by SOX11 targeting.	Diagnosis	40 patients 4 cell lines	China	2018	Wang [107]
miR-423-5p	SOX4	Circ_0039411 increased PTC cell migration through the regulation of miR-423-5p/SOX4 axis.	Diagnosis	51 patients 2 cell lines	China	2021	Wen [108]
miR-25 and miR-30d	EZH2	MiR-25 and miR-30d had a crucial role in ATC progression through EZH2 targeting.	Diagnosis	11 patients 3 cell lines	France	2012	Esposito [109]
miR-524-5p	FOXE1	MiR-524-5p modulated the PTC cell invasion, migration, and proliferation through FOXE1 suppression.	Diagnosis	57 patients 3 cell lines	China	2019	Liu [110]
miR-206	MRTFA	MiR-206 suppressed ATC cells migration through MRTFA targeting.	Diagnosis	19 patients 2 cell lines	China	2015	Zhang [111]
miR-637	LMO4	Knockdown of circLDLR reduced PTC cell migration, while promoted apoptosis by miR-637/LMO4 axis.	Diagnosis	45 patients 2 cell lines	China	2021	Jiang [112]
Membrane Associated And Extra Cellular Factors							
miR-206	RAP1B	MiR-206 significantly decreased TPC-1 migration through RAP1B targeting.	Diagnosis	60 patients 5 cell lines	China	2019	Wang [113]
miR-126-3p	SLC7A5 and ADAM9	MiR-126-3p suppressed TC cell proliferation, migration, and distant metastasis through SLC7A5 and ADAM9 targeting.	Diagnosis and prognosis	496 patients 3 cell lines	USA	2015	Xiong [114]
miR-30a	LOX	MiR-30a inhibited ATC cell migration and invasion via LOX suppression.	Diagnosis and prognosis	14 patients 4 cell lines	USA	2015	Boufraqech [115]
MiR-613	TAGLN2	MiR-613 inhibited PTC cell invasion and EMT via TAGLN2 targeting.	Diagnosis and prognosis	107 patients 3 cell lines	China	2021	Huang [116]
miR-1231	GPX4	CircKIF4A promoted the PTC cell migration while inhibited ferroptosis by miR-1231 sponging and subsequent GPX4 up regulation.	Diagnosis	3 cell lines	China	2021	Chen [117]
Apoptosis And Protein Degradation							
miR-618	XIAP	MiR-618 repressed ATC cell migration and invasion by targeting XIAP.	Diagnosis and prognosis	2 cell lines	China	2014	Cheng [118]
miR-15	BCL-2	MiR-15 inhibited MDA-T35 cells migration through BCL-2 suppression.	Diagnosis	4 cell lines	China	2019	Lu [119]
miR-34a-5p	TP73	CASC7 suppressed the PTC cell proliferation and migration by miR-34a-5p sponging and TP73 up regulation.	Diagnosis	30 patients 2 cell lines	China	2021	Sun [120]
miR-214	PSMD10	MiR-214 suppressed PTC cell migration and EMT via PSMD10 targeting.	Diagnosis and prognosis	30 patients 2 cell lines	China	2018	Liu [121]
miR-4319	SMURF1	MiR-4319 repressed the TC cell migration and EMT through SMURF1 targeting.	Diagnosis and prognosis	56 patients 5 cell lines	China	2020	Bian [122]
MiR-192-5p	SH3RF3	MiR-192-5p reduced PTC cell migration and EMT, while increased apoptosis by SH3RF3 targeting.	Diagnosis	20 patients 3 cell lines	China	2021	Fu [123]

### MiRNAs involved in regulation of PI3K/AKT signaling

**Fig. 1:**
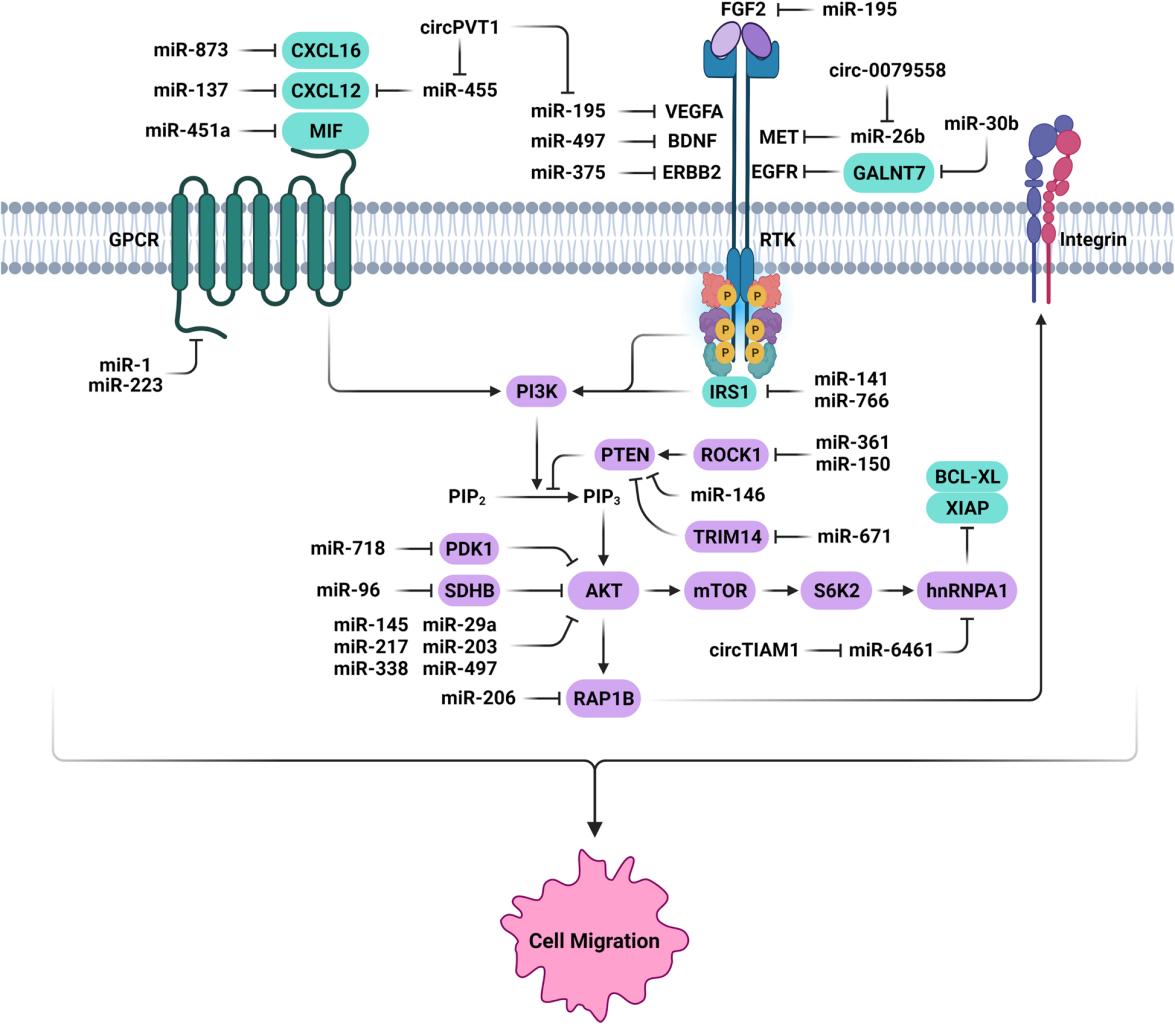
Role of miRNAs in thyroid tumor cell migration via regulation of PI3K/AKT and chemokine signaling pathways. (Created with BioRender.com)

Cell migration is a critical process in normal and tumor cells. The PI3K kinases regulate cell migration through directly or through activation of other signaling pathways [124]. MiRNAs have critical roles in regulation of thyroid tumor cell migration through PI3K/AKT signaling (Fig. 1). AKT is a component of the PI3K/AKT pathway involved in cell proliferation, programmed death, migration, invasion, and metabolism [125–128]. A significant inverse correlation has been reported between miR-497 and AKT3 in PTC. MiR-497 also inhibited PTC cells migration and invasion through AKT3 suppression [41]. The tumor-suppressive role of miR-145 has been suggested in various cancers. Moreover, miR-145 has been revealed to participate in cell differentiation and proliferation [129, 130]. It has been reported that there were significant reduced levels of miR-145 expressions in PTC, PDTC, and ATC compared with normal thyroid tissue. MiR-145 targeted the AKT3, N-cadherin, and HIF1α during thyroid cancer progression. Although, there were increased serum levels of miR-145, it was under expressed in tissue samples [42]. MiR-217 expression has been also revealed to be significantly reduced compared with normal tissues in TC patients. The miR-217 down regulation was also associated with clinical stage and lymphatic metastasis. MiR-217 also suppressed the growth of the tumors in vitro and in vivo through the AKT3 targeting in TC [43]. MiR-203 has a cardio protective role through inhibiting the PI3K/AKT in diabetic cardiomyopathy [131]. It has been reported that the miR-203 up regulation and AKT3 down regulation reduced tumor cell migration, increase E-cadherin, and reduce vimentin expression levels in PTC cells [44]. A significant miR-338-3p up regulation in TC tissues in comparison with normal margins was inversely correlated with the clinical stage and lymphatic metastasis. The miR-338-3p also significantly suppressed the cell migration of TC cells and repressed tumor development in vivo by targeting AKT3 [45]. Another study has been observed that there was miR-29a down regulation in PTC tissues which was inversely correlated with TNM stage, tumor size, and lymphatic metastasis. It also suppressed PTC cell migration and invasion by targeting AKT3 [46]. AKT2 is an oncogenic serine/threonine kinase with SH2 domains capable of phosphorylating downstream targets. The up regulation of Let-7a expression has been revealed to significantly inhibit the PTC migration and invasion by targeting AKT2. There was also Let-7a down regulation in PTC compared with healthy tissues [47]. AKT is a member of AGC family regulated by PDPK1 that is involved in insulin and growth factors signaling [132]. PDPK1 has a significant role in regulation of AGC protein kinases family, which controls cell proliferation, growth, and metabolism [133]. MiR-718 is a critical negative regulator for the PTC cell proliferation and migration through PDPK1 targeting [48].

PI3K family is involved in regulation of cellular growth, proliferation, and glucose homeostasis. MiR-125b has been reported to be significantly down regulated in the ATC tissues and cell lines. It directly targets PIK3CD and inhibits the migratory and invasive capacity of tumor cells via PI3K-Akt-mTOR cascade. While up regulated miR-125b significantly inhibited the invasive and migratory behavior of cells, its knockdown promoted the invasion and migration of ATC cells [49]. Another group has been shown that there was miR-363-3p under expression in TC tissues and cell lines which was inversely associated with clinical stage and lymph node metastases. It significantly repressed migratory and invasive behaviors of PTC cells and prevented in vivo tumorigenesis. There was a significant negative relationship between PIK3CA and miR-363-3p expression, indicating PIK3CA as a target of miR-363-3p in PTC [50]. PPM1E is a serine/threonine-protein phosphatase that has important role in cell proliferation by regulation of AMPK-mTOR signaling [134]. There was LINC01087 up regulation and miR-135a-5p down regulation in TC tissues and cell lines compared with normal margins and cells. LINC01087 induced TC cell migration via miR-135a-5p/PPM1E axis [51]. HnRNPA1 as a substrate for S6K2 in AKT/mTOR signaling has a pivotal anti-apoptotic function [135]. CircTIAM1 induced PTC cell migration through the miR-646/HNRNPA1 axis [52]. GALNT7 induces cell proliferation and migration via the activation of EGFR/PI3K/AKT pathway. MiR-30b-5p down regulation was correlated with poor clinicopathological features in PTC. MiR-30b-5p inhibited PTC cell proliferation and migration by GALNT7 targeting [53].

MiR-141 acts as an anti-oncogene by targeting EphA2, E2F3, ZEB2, Tiam1, KEAP1, and SOX17 [136–141]. IRS2 belongs to the IRS protein family that interacts with the SH2-domain-containing proteins, primarily PI3K during insulin signal transduction [142, 143]. It is a multifunctional oncogene that regulates cell proliferation, invasion, and EMT [144–146]. It has been shown that miR-141 inhibited cell migration and invasive capability of thyroid tumor cells. It also triggered cell death, and repressed tumor growth by IRS2 targeting. Moreover, miR-141 down regulation was significantly correlated with TNM stage and lymph node involvement [54]. Another study also showed that the miR-766 inhibited the malignant activity of PTC cells through IRS2 suppression. There was a significant miR-766 down regulation in PTC tissues and cell lines which was correlated with TNM staging and lymph node involvement [55].

Succinate dehydrogenase (SDH) is a crucial enzyme in the mitochondrial citric acid cycle [147–149]. This enzyme is involved in succinate to fumarate oxidation and electron transport [150, 151]. SDHB deregulation is involved in oxidative phosphorylation in several cancers [147, 152, 153]. The SDHB dysfunction elevates TGFβ-mediated colorectal cancer invasion and metastasis via the SNAIL1-SMAD3/4 transcriptional repressor complex [154]. It has been reported that there was increased levels of miR-96-3p expressions in PTC tissues in comparison with normal margins which was associated with advanced tumor stage. The miR-96-3p up regulation increased tumor cell invasion and TC metastasis by regulating SDHB/AKT/mTOR pathway [155]. It has been observed that there were decreased levels of miR-215 expressions in PTC tissues that were correlated with tumor size and invasion. In vivo and in vitro studies have also shown that the miR-215 up regulation significantly repressed the proliferation and metastasis of PTC cells through targeting ARFGEF1. MiR-215 suppressed PTC metastasis by regulating the AKT/GSK-3β/Snail cascade and ARFGEF1 targeting [56].

MIF is a pivotal pro-inflammatory cytokine during tumor progression and growth by AKT activation [156]. It has been shown that there was miR-451a down regulation in PTC samples which was significantly correlated with the aggressive PTC and advanced TNM stage. There was also an inverse association between the levels of MIF and miR-451a expressions that suggested the MIF as the target of miR-451a [57]. The miR-146b is correlated with tumor aggressiveness and poor prognosis of PTC [157]. MiR-146b up regulation enhances cell migration, EMT, and metastasis [158]. It is involved in regulation of PTEN/PI3K/AKT pathway during thyroid tumor aggressiveness. PTEN suppression by miR-146b revealed some information about the processes and positive feedback loops mediating the PI3K pathway activation in thyroid tumorigenesis. MiR-146b also reduced pSTAT3 and E-cadherin levels. TWIST as an important EMT factor, may mediate the effect of miR-146b on invasion and migration since its expression level is up regulated in cells with miR-146b up regulation. In contrast, E-cadherin is down regulated in cells with miR-146b overexpression [58]. TRIM14 is involved in regulation of PI3K/AKT pathway by the PTEN inhibition [159]. MiR-671-5p inhibited the PTC cell migration by TRIM14 targeting [59]. CircWDR27 increased PTC cell migration by regulation of the miR-215-5p/TRIM44 axis [60]. There was an inverse association between the levels of LINC00958 and miR-627 expressions in PTC samples. LINC00958 increased PTC cell migration by miR-627 sponging and TRIM44 up regulation [61].

### MiRNAs involved in regulation of WNT and Hippo signaling pathways

**Fig. 2:**
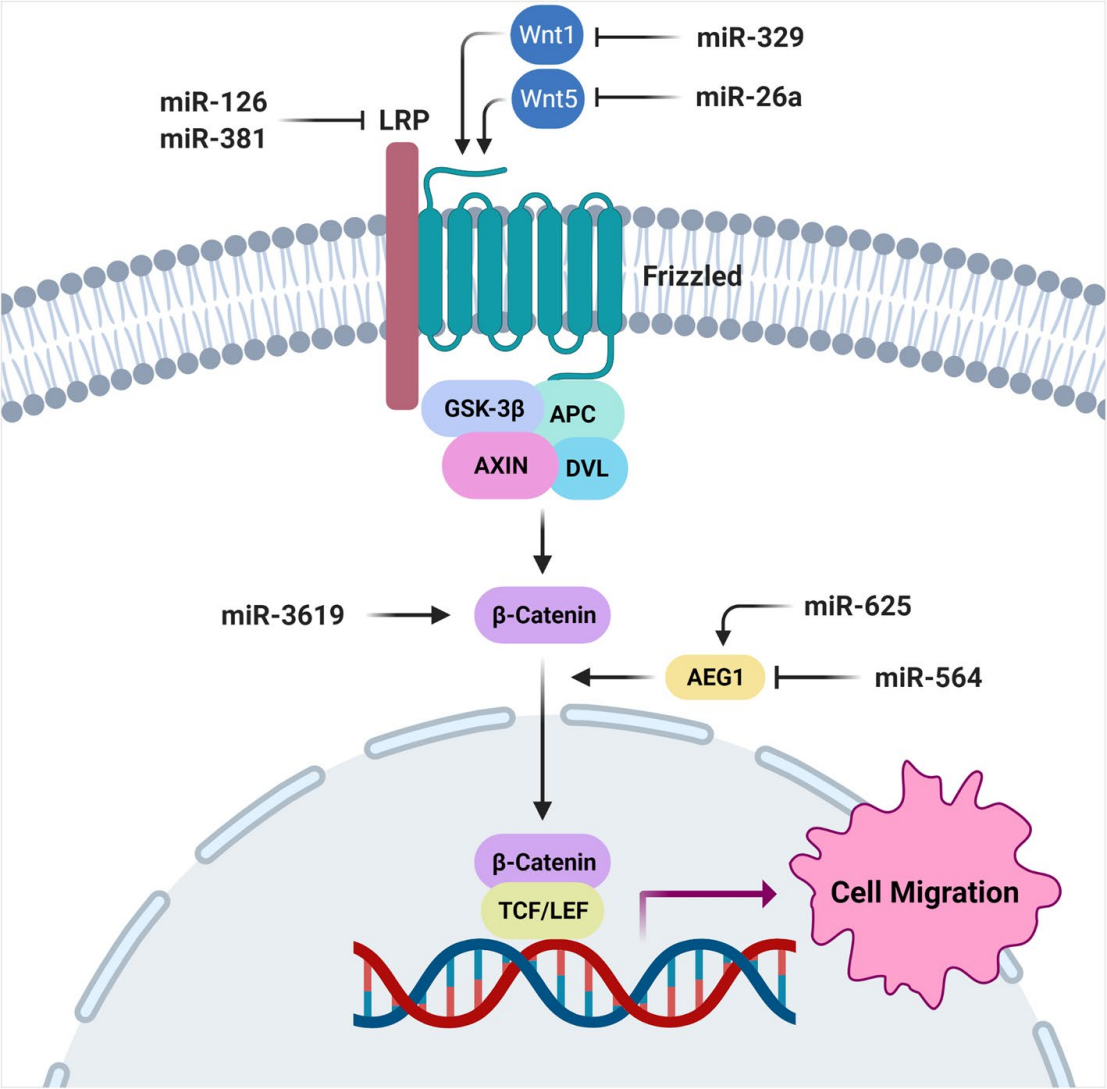
Role of miRNAs in thyroid tumor cell migration via regulation of WNT signaling pathway. (Created with BioRender.com)

WNT/b-catenin is a pivotal signaling pathway involved in cell proliferation, polarity, embryogenesis, and tumor progression [160–162]. It participates in the neoplastic transformation from low grade to high-grade or ATC with poor prognosis [163]. MicroRNAs are critical factors in thyroid tumor cell migration by regulation of WNT signaling pathway (Fig. 2). It has been revealed that there were correlations between miR-3619-3p up regulation, extra thyroidal extension, and lymphatic metastases in PTC tissues. The miR-3619-3p also promoted the migratory and invasive capability of PTC cells through increased b-catenin mRNA stability [62]. LRP6 is a co-receptor of the WNT signaling, which induces the WNT target genes by increased rate of b-catenin nuclear transport [164, 165]. It has been shown that the miR-126 up regulation suppressed PTC cells migration and in vivo tumor growth through LRP6 suppression. There was also a significant miR-126 down regulation in PTC tissues and cell lines which was significantly associated with lymph node involvement, tumor size, and TNM stage. Moreover, suppression of LRP6 by miR-126 resulted in down regulation of CCND1, MMP7, and C-myc [63]. Another group observed that the miR-381-3p was associated with PTC metastasis through LRP6 down regulation. There were also significant decreased miR-381-3p expression in PTC tissues and cell lines [64]. WNTs are secretory glycoproteins with pivotal roles during cell differentiation, proliferation, and migration [166]. WNT5A is a positive regulator of classical or non-classical WNT pathways. It has been reported that there was a miR-26a-5p down regulation in PTC cells and tissues compared with healthy thyroid cells and tissues. It also inhibited the PTC cell growth, invasion, and metastasis via WNT5a suppression [65]. MiR-329 is reported to be down regulated in PTC samples and cell lines compared with normal margins and healthy cell lines. It also inhibited the cell migration, invasion, and in vivo tumor growth via WNT1 suppression. Moreover, there was an inverse correlation between the levels of miR-329 and WNT1 mRNA expressions in PTC samples [66].

Forkhead Box N3 (FOXN3) has a crucial role as a transcriptional repressor in cell cycle regulation and carcinogenesis [167]. It has been reported that the miR-574-5p mediated TC cell proliferation and migration through the Wnt/β-catenin pathway by targeting FOXN3. MiR-574-5p silencing prevented the cell proliferation and migration, while enhanced apoptosis in TC [67]. SCAI is a tumor suppressor that inhibits cell migration through β1-integrin and WNT signaling suppression [168, 169]. Abnormal miR-1270 up regulation has been revealed in human PTC cell lines and tumors. Down regulation of miR-1270 suppressed the PTC tumor cells migration and in vivo transplantation. It has been shown that the miR-1270 was involved in PTC progression through SCAI targeting [68]. MiR-625-3p is an oncogene in various cancers that induce the tumor cell proliferation, migration, and drug tolerance [170, 171]. AEG-1 is correlated with PTC metastasis by up regulations of MMP2 and MMP9 [172]. It has been reported that there was miR-625-3p up regulation in PTC tissues compared with normal margins. MiR-625-3p up regulation enhanced the migratory and invasive capability of SW579 and TPC-1 cell lines. Besides, AEG-1 was up regulated by miR-625-3p in SW579 and TPC-1 cell lines. AEG-1 up regulation also enhanced cell migration, and activated the WNT and JNK signaling pathways. Moreover, there were significant up regulations of BAX, CASP3, and CASP9 following miR-625-3p suppression in SW579 and TPC-1 cells. Therefore, miR-625-3p enhanced TC cell migration and invasion by AEG-1 up regulation and triggering WNT and JNK pathways [69]. Another group showed the negative role of miR-564 on PTC migration and invasion via AEG1 inhibition. There was also miR-564 under expression in PTC tissues and cell lines which was significantly correlated with lymph node involvement and the TNM stage [70].

YAP1 (yes-associated protein 1) is one of the key effectors of Hippo signaling pathway which is involved in regulation of OCT4 and SOX2 to promote self-renewal capability [173]. It has been reported that there was miR-205 down regulation in PTC samples. It also inhibited the Hippo signaling cascade by targeting YAP1 and suppressed TC cell migration and invasion [71]. SNHG15 is a competitively endogenous RNA (ceRNA) that regulates the Hippo signaling pathway through miR-200a-3p sponging in PTC [174]. It has been shown that there was miR-510 up regulation in TC tissues and cell lines compared with the healthy thyroid tissues and cell lines. There was an inverse association between SNHG15 and miR-510-5p expression. The miR-510-5p also promoted the TC cell migration and invasion via SNHG15 targeting. Moreover, miR-510-5p up regulation has been reported in advanced stage tumors with lymphatic metastasis [15]. CRABP2 is a positive regulator of EMT by inhibition of Hippo pathway [175]. LINC01816 increased TC cell migration and EMT process via miR34c5p/CRABP2 axis [72].

### MiRNAs involved in regulation of TGFβ signaling pathway

**Fig. 3:**
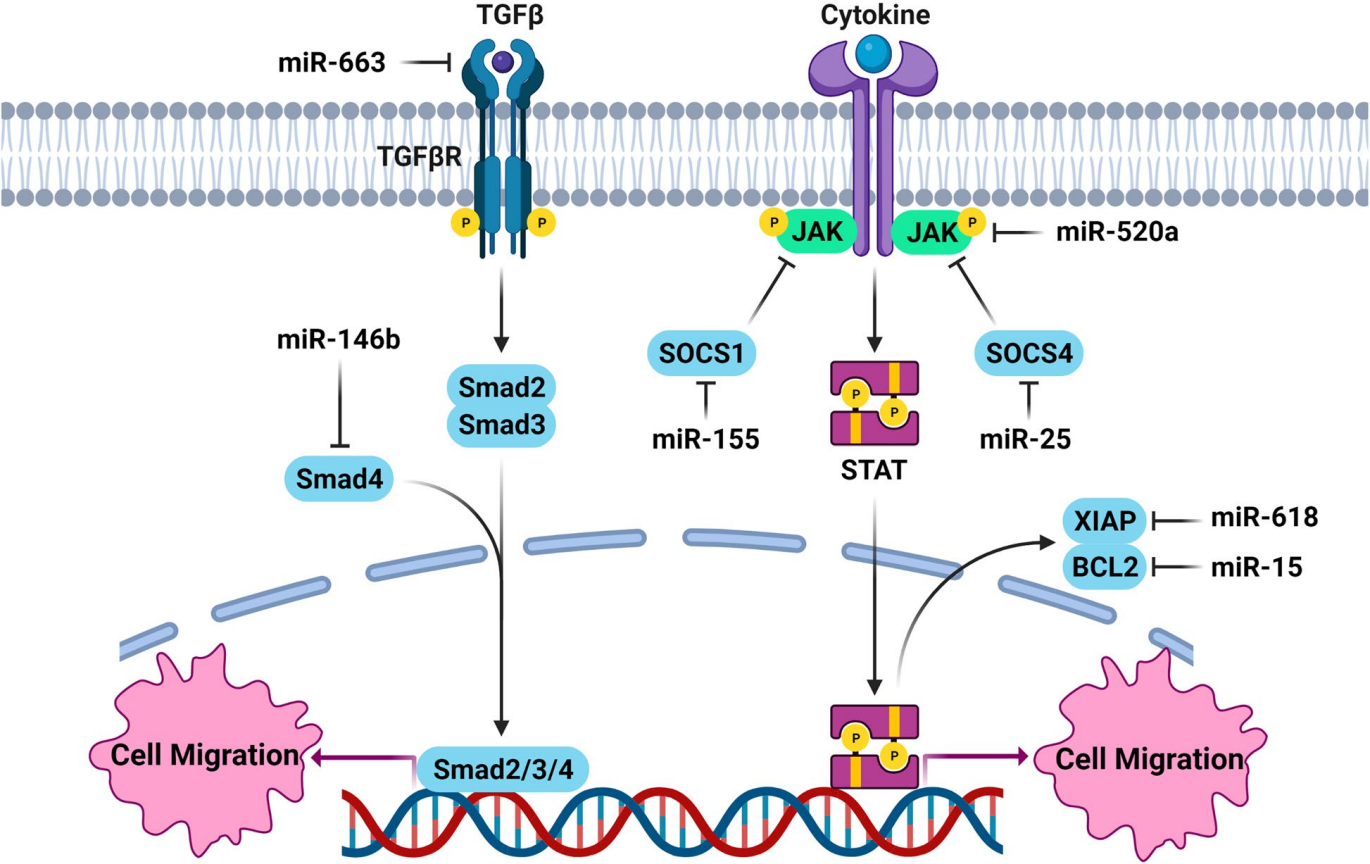
Role of miRNAs in thyroid tumor cell migration by regulation of TGFβ and cytokine signaling pathways. (Created with BioRender.com)

TGFβ1 is a cytokine that functions as a tumor suppressor in the early stages of tumor progression and as an oncogene during advanced stages of tumors [176, 177]. It regulates the tumor cell migration and invasion by Smad-dependent or Smad-independent signaling pathways [176, 178]. It has also a critical role in EMT, characterized by E-cadherin and cytokeratin down regulations and increased expressions of N-cadherin and vimentin [176, 179, 180]. MicroRNAs are involved in TC cell migration through the regulation of TGFβ signaling (Fig. 3). It has been reported that there was miR-663 down regulation in PTC cases which was correlated with extra thyroidal extension, age, tumor size, and poor prognosis. It also reduced PTC cell invasion, migration, and EMT via TGFβ inhibition. The overexpressed-miR-663 PTC cell lines had E-cadherin over expression, while N-cadherin, vimentin, MMP-2, and MMP-9 down-regulations [73]. MiR-146b-5p is involved in regulation of TGF-b signal transduction through suppressing SMAD4 in PTC [181] and enhancing tumor metastasis through ZNRF3 targeting [158]. It has been shown that there was miR-146b-3p up regulation in PTC samples. It also promoted cell migration and invasion via NF2 suppression [182]. Another study has been shown that miR-146b-5p was involved in tumor cell migration through SMAD4 targeting in PTC [74]. PARD protein family regulates the polarity, migration, and cell proliferation [183]. Cell polarity disruption in various cancers has been suggested to be influenced by PARD impairment. Interaction of PARD3 and TGF-β may alter the PARD complex, leading to a neoplastic transformation [184]. PARD3 prevented the actin polymerization triggered by Tiam1-Rac1 and thus stabilized cell-cell contacts and repressed the tumor cells migration. It has been reported that miR-483 up regulated the Tiam1-Rac1 signals through PARD3 down regulation. Although, PARD3 inhibited the TGF-β1-mediated EMT and invasion, it’s down regulation via miR-483 stimulated Tiam1-Rac1 and facilitated EMT and migration of TC cells. Moreover, MiR-483 and PARD3 have been reported to be up regulated and down regulated in thyroid tumor samples, respectively [75].

### MiRNAs involved in regulation of cytokines

Cytokines have a pivotal role in TC cell migration and metastasis that can be regulated by the microRNAs (Fig. 3). IL-22 is a member of IL-10 family cytokines that is mainly produced by activated Th17 or Th22 cells [185–187]. It is involved in antimicrobial defense in the gastrointestinal tract, tissue regeneration, and injury protection [188–190]. It is correlated with tumor progression and metastasis in breast, lung, and gastric cancers [191–193]. SOX17 belongs to the SOX family of transcription factors and has a crucial role in human tumorigenesis [194]. It has been found that the IL-22 induced miR-595 expression which resulted in SOX17 down regulation and increased PTC cell migration [195]. The chemokine superfamily is small and structurally related proteins, capable of binding and interacting with G-protein coupled receptors, leading to cytoskeletal rearrangement and directional cell migration [196]. SOCS1 is a negative regulator of cytokine signal transduction [197]. The miR-155 up regulation was reported among ATC tissues. There were also correlations between miR-155 expression and extra thyroidal invasion. Moreover, Overexpression of miR-155 enhanced tumor progression by SOCS1 suppression [76]. IL-23 is a type 1 cytokine which is a member of the IL-6 superfamily [198]. It has been shown that IL-23 regulated the migratory and invasive behavior of TC cells through miR-25/SOCS4 signaling. MiR-25 enhanced the TC migration via targeting SOCS4. Moreover, it regulates the expression of IL-23-associated SOCS4 in TC cells [77].

Chemokines and their receptors have critical roles in tumor cell migration and metastasis [199]. MicroRNAs are involved in regulation of TC cell migration by chemokine targeting (Fig. 1). It has been reported that there was miR-126 down regulation in TC tissues and cell lines. It also reduced tumor cell migration and invasion via CXCR4 targeting [78]. DUXAP8 induced PTC cell migration and proliferation through the regulation of miR-223-3p/CXCR4 axis [79]. CXCL12 is a potent chemo attractant for hematopoietic cells and essential for the migration of tumor cells [200]. It binds with its CXCR4 receptor to activate the PI3K/AKT and ERK signaling pathways leading to cell migration [201]. A significant down regulation of miR-137 has been revealed in PTC tissues which were negatively associated with tumor TNM classification and lymph node metastasis. It also inhibited PTC cell migration by targeting CXCL12 [80]. There was an inverse association between the levels of miR-455-5p and circPVT1 that was correlated with MTC prognosis. MiR-455-5p suppressed EMT and invasion in MTC cells by CXCL12 targeting [81]. It has been observed that there were decreased levels of miR-1 expressions in benign and malignant thyroid neoplasms compared with healthy thyroid tissue. MiR-1 regulated the TC cell migration through CXCR4, SDF-1, and MET suppression [82]. CXCL16-CXCR6 interaction is crucial during tumor progression of the aggressive cancers [202]. It has been reported that there was miR-873-5p down regulation and CXCL16 up regulation in PTC samples that introduced CXCL16 as a target of miR-873-5p. MiR-873-5p also inhibited PTC cells migration by targeting CXCL16 [83].

### MiRNAs involved in regulation of kinases

BDNF is a member of neurotrophin family of tyrosine kinase receptors which is involved in regulation of PI3K/AKT, RAS/ERK, PLC/PKC, and JAK/STAT signaling pathways during tumor progression [203]. It has been shown that there was miR-497 down regulation in TC samples compared with normal margins which was inversely associated with lymph node involvement and advanced clinical stage. It also suppressed cells colony-forming, migration, and in vivo tumor growth through BDNF targeting and PI3K-AKT inhibition [84]. MET belongs to the tyrosine kinase receptor protein family that regulates cell growth. It has been reported that there were circ-0079558 and MET up regulations in PTC tissues and cell lines. Circ-0079558 promoted the PTC cell proliferation and migration, while reduced apoptosis via miR-26b-5p/MET/AKT axis [85]. ERBB2 is a tyrosine kinase receptor associated with tumor progression [204]. It has been reported that the miR-375 was down regulated in PTC tissues and cell lines. It also suppressed the proliferative capability of PTC cell lines by apoptosis induction. Moreover, miR-375-mimic transfection significantly reduced PTC cell migration and invasion through ERBB2 suppression [86]. FGF2 is a ligand of FGFR that is associated with tumor cell migration and invasion [205]. It has been reported that there were significant miR-195 down regulation in PTC tissues and cell lines. It also suppressed PTC cell migration through CCND1 and FGF2 suppression. Moreover, miR-195 enhanced the phosphorylation of β-catenin which inhibited WNT signaling in PTC [206]. ATC is an aggressive, malignant neoplasm, distinguished by poor prognosis [207–209]. EGFR is a tyrosine kinase up regulated in the majority of ATC samples [210]. The EGF is produced by the thyroid gland and promotes the migratory and invasive behaviors of thyroid cancer cells [209, 211–214]. It has been shown that the EGF/EGFR activation reduced E-cadherin and up regulated vimentin. EGF/EGFR pathway modulated the aggressive behaviors of SW1736 cells by regulating miR-200 in which, EGF reduced miR-200, increased Rho/Rock activity, and increased EMT in TC cells [215]. VEGFA is a tyrosine kinase receptor involved in angiogenesis. CircPVT1 promoted the PTC cell migration by miR-195 sponging and subsequent VEGFA up regulation [87]. Janus kinases (JAK) are a class of tyrosine kinases involved in cell growth, differentiation, and apoptosis. It has been reported that the miR-520a-3p inhibited the JAK-STAT signaling by JAK1 suppression which inhibited EMT and migration of PTC cells. The miR-520a-3p up regulation or JAK1 knockdown resulted in under expression of JAK1 and EMT-linked markers such as E-cadherin and vimentin in PTC tissues [88].

ROCK1 is a serine/threonine kinase belonging to the Rho-associated kinase family (ROCK) that participates in various cellular processes such as mitochondrial fission, cell signaling, and cytoskeletal organization [216, 217]. It has been shown that there were significant correlations between miR-361-5p down regulation, lymphatic metastasis, and TNM stage in PTC cases. The miR-361-5p up regulation also inhibited the migration and invasion of TPC-1 cells. Besides, miR-361-5p diminished in vivo tumor development in PTC xenografts. Moreover, the miR-361-5p under expression was correlated with poor prognosis in PTC cases. There was an inverse correlation between the expression level of ROCK1 and miR-361-5p which showed miR-361-5p prevented PTC cells migration by ROCK1 targeting [89]. Another group also showed that the miR-150-5p inhibited PTC cell migration through ROCK1. There was a reduced levels of miR-150 expression in PTC tissues and cell lines compared with controls. The miR-150-5p down regulation was also correlated with TNM stages and lymph node metastasis [90].

Sphingolipids consist of a wide variety of hydrophobic molecules including Ceramide, Sphingoid, Ceramide Phosphates, and Sphingoid Base Phosphates [218]. The proliferative and invasive behavior are associated with dynamic equilibrium between ceramide phosphates and sphingoid phosphates [219]. Sphingosine kinases (SPHKs) involve two different variants, including SPHK1 and SPHK2 [220, 221]. SPHK1 catalyzes S1P (Sphingosine 1-phosphate) formation from pro-apoptotic sphingolipid [219]. It has been shown that there were decreased levels of miR-128 expressions in PTC, FTC, and TC cell lines. The miR-128 reduced TC cell migration and invasion via targeting SPHK1. Besides, the up regulation of miR-128 significantly induced cell death in TC cell lines through CASP-3 up regulation. MiR-128 up regulated and down regulated the E-cadherin and N-cadherin, respectively [91]. Another group showed that the miR-577 up regulation suppressed PTC cells migration and invasion through SPHK2 suppression. There were also significant miR-577 down regulations in PTC tissues and cell lines [92]. MiR-613 significantly inhibited the PTC cell migration, invasion, and in vivo growth by targeting SPHK2. Moreover, there was miR-613 down regulation in PTC cell lines and tissues [93].

CARMA1 is a member of membrane-associated guanylate kinase family that functions as membrane scaffolds. It has a vital function in NF-kB regulation which is induced by antigen-receptor in T and B lymphocytes [222, 223]. CARMA1 is phosphorylated by protein kinase C-theta (PKC-θ) following T cell receptor and CD28 binding, leading to the formation of an active complex that consists of CARMA1 as well as downstream signaling scaffolding adaptor proteins, including BCL10 and MALT1 [224, 225]. Subsequently, these molecules are recruited into the lipid micro-domains at the immune synapse, which results in activating KK complex and NF-kB [226]. It has been reported that the CARMA1 was targeted and down regulated by miR-539, thereby inhibiting thyroid tumor cell metastasis. Therefore, miR-539 is a modulator of TC cell migration and invasion by targeting CARMA1 [94].

MAPK1 is a serine/threonine kinase involved in MAP/ERK signaling pathway. MiR-675 down regulation has been observed in PTC tissues and cell lines that were significantly associated with lymphatic metastases and the TNM stage. It also inhibited PTC cell migration and in vivo growth through MAPK1 regulation [95]. The p21-activated kinase (PAK1) is also a serine/threonine kinase that regulates cytoskeletal remodeling, directional motility, metastasis, and cell cycle progression. It has a pivotal role in interaction between Rho GTPases and cytoskeletal reorganization [227]. The miR-7 down regulation has been shown in TC tissues which were correlated with stage. It also inhibited TC cells migration and invasion through targeting PAK1 [96]. RAC1 as a Rho GTPase is involved in cell proliferation, adhesion, and migration [228, 229]. RAC1/PAK1 signaling is associated with high glucose-induced podocyte EMT through β-catenin and Snail induction [230]. It has been reported that there was miR-101 down regulation in PTC samples compared with normal margins. It also repressed PTC cell invasion and migration through RAC1 targeting [97].

### MiRNAs associated with transcriptional regulation and transcription factors

**Fig. 4:**
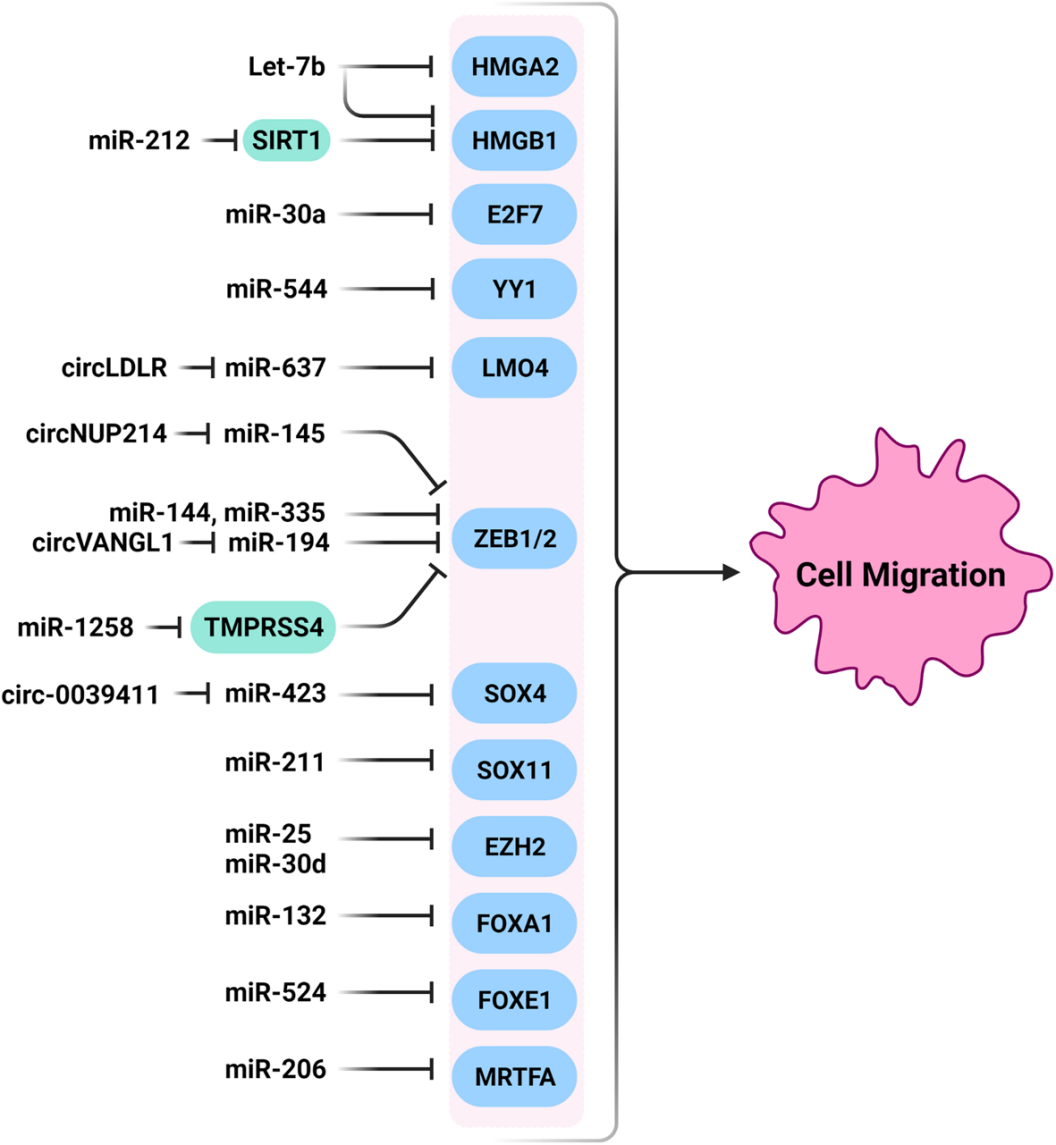
Role of miRNAs in thyroid tumor cell migration and EMT by regulation of transcription factors. (Created with BioRender.com)

Signaling pathways mainly exert their role in regulation of tumor cell migration by specific transcription factors. It has been shown that the miRNAs have key roles in thyroid tumor cell migration and EMT by regulation of transcription factors (Fig. 4). High-mobility group-A2 gene (HMGA2) is a non-histone chromatin protein that regulates the chromatin remodeling to induce or suppress transcriptional enhancer activity through binding with AT-rich DNA sequences [231]. HMGA2 has a vital role in tumor growth through regulation of EMT and metastasis [232, 233]. It has been reported that there was significant decreased levels of let-7b in PTC cell lines and tissues. Let-7b inhibited PTC cell migration and invasion through HMGA2 targeting [98]. HMGB1 is also an important chromatin binding protein that interacts with transcription factors and histones to regulate the chromatin remodeling and transcription [234]. It has been reported that the let-7e suppressed PTC migration through HMGB1 targeting [99]. SIRT1 is a NAD-dependent deacetylase involved in regulation of cell-cycle, death, and tumor metastases through MYC, TP53, and NF-κB [235, 236]. It inhibits ovarian tumor cell migration and invasion via HMGB1 deacetylation and down regulation [237]. It has been reported that there was miR-212 down regulation in TC tissues and cell lines which was associated with lymphatic metastasis and clinical stage. It also inhibited cell proliferation, migration, and in vivo growth through SIRT1 targeting [100].

ZEB1 and ZEB2 are two E-box-binding transcription factors associated with tumorigenesis [238]. These transcription factors are crucial regulators of the transcription repressor network, which inhibit multiple key epithelial polarity regulators implicated in EMT and invasion [239, 240]. It has been reported that there was miR-144 down regulation in TC. The miR-144 suppressed migration of thyroid tumor cells through targeting ZEB1 and ZEB2 [101]. Another reports also showed that the miR-429 and miR-335 suppressed TC cell migration through ZEB1 and ZEB2 targeting, respectively. Moreover, there were miR-429 and miR-335 down regulations in TC samples and cell lines [102, 103]. Non-coding RNAs (ncRNAs) include long non-coding RNAs (lncRNAs), miRNAs, and Circular RNAs (circRNAs) [241–243]. Intracellular circRNAs suppress the miRNA by sponging and binding to the microRNA response element (MRE) [244]. It has been observed that there was circNUP214 up regulation of in PTC in comparison with normal margins. It also induced tumor cells migration and invasion through miR-145 sponging which resulted in ZEB2 up regulation [245]. There was significant circVANGL1 up regulation in PTC tissues that was associated with poor prognosis. CircVANGL1 promoted PTC cell migration and EMT process miR-194 sponging and ZEB1 up regulation [104]. TMPRSS4 is a serine protease associated with tumor cells migration and adhesion by EMT promotion through SIP1/ZEB2 induction [246]. There were reduced levels of miR-1258 in PTC cell lines. The miR-1258 suppression also increased the PTC cell migration and invasion via TMPRSS4 regulation [247].

E2F7 has been widely recognized as a transcriptional repressor of promoters with E2F consensus sequence [248]. It acts as a critical component of the inhibitory loop which is needed to switch off the transcription of E2F-driven G1/S target genes during cell cycle [249]. It has been shown that there was miR-30a down regulation in PTC cells. It also inhibited the PTC cell migration by targeting E2F7 [105]. Yin Yang 1 (YY1) is belonged to the GLI-Kruppel family of zinc-finger transcription factors involved in various cellular processes such as embryogenesis, cell proliferation, cell death, and DNA repair [250]. It has been reported that there was miR-544 down regulation in ATC tissues. It also suppressed tumor metastasis by targeting YY1. The YY1 knockdown repressed cell viability and proliferation and substantially reduced ATC cell migration and invasion [106]. SOX11 is a developmental transcription factor involved in neurodevelopment, neural cell survival, and neurite outgrowth [251]. It has been reported that the miR-211-5p suppressed TC cells migration and invasion by SOX11 targeting [107]. There was circ_0039411 up regulation in PTC tissues and cell lines in comparison with normal margins and cells. Circ_0039411 increased PTC cell migration through the regulation of miR-423-5p/SOX4 axis [108].

EZH2 belongs to the Polycomb-group (PcG) family that is involved in transcriptional repression by histone methylation and chromatin condensation. MiR-25 and miR-30d prevented ATC cell proliferation and colony formation through G2/M arrest. It has been observed that there were inverse correlations between EZH2 protein levels and miR-25 and miR-30d expressions in ATC specimens. MiR-25 and miR-30d had a crucial role in ATC progression through EZH2 targeting [109]. FOXA1 as a transcriptional activator is belonged to the forkhead family of DNA-binding proteins. It has been shown that there were reduced expression levels of miR-132 in TC tissue samples and cell lines, in which the miR-132 inhibited TC cell migration and invasion by suppressing FOXA1 [252]. FOXE1 is also a member of forkhead transcription factors that acts as an important factor during thyroid morphogenesis. It enables polarized epithelial cells to transfer into the cellular matrices, which is required for the tumor invasion and metastasis. It has been observed that the miR-524-5p modulated the PTC cell invasion, migration, and proliferation through FOXE1 and ITGA3 suppression [110].

MRTFA is a transcriptional coactivator involved in cytoskeletal dynamics during cell migration. It has been reported that there was MRTFA up regulation in ATC tissues in comparison with primary tumor tissue. MiR-206 suppressed ATC cells migration through MRTFA targeting [111]. LMO4 is a transcription factor involved in regulation of cell proliferation. There was significant circLDLR up regulation in PTC tissues and cell lines. Knockdown of circLDLR reduced PTC cell migration, while promoted apoptosis by miR-637/LMO4 axis [112].

### MiRNAs involved in regulation of membrane associated and extra cellular factors

RAP1B is a member of RAS-family GTPases that has a vital role in cell proliferation, migration, and invasion. As a membrane associated GTPase, it regulates integrin-mediated signaling. It has been reported that there were significant reduced levels of miR-206 expressions in TC tissues and cell lines compared with healthy tissues. The miR-206 also significantly decreased TPC-1 migration through RAP1B targeting [113]. SLC7A5 is a membrane transporter involved in thyroid hormones transportation [253]. ADAM9 belongs to the ADAM family, which has a crucial role in the shedding of membrane-bound proteins [254]. It has been shown that the miR-126-3p suppressed TC cell proliferation, migration, and distant metastasis through SLC7A5 and ADAM9 targeting. Moreover, there was a significant correlation between miR-126-3p down regulation, aggressiveness, tumor size, local invasion, and recurrence [114].

ATC has a survival rate of fewer than six months following diagnosis and is one of the deadliest malignancies [255, 256]. More than two-thirds of ATC patients suffer from locally advanced and metastatic ATC [256, 257]. LOX protein belongs to the amine oxidases family involved in tumors progression [258]. As an ECM remodeling enzyme, LOX is essential for the lysine deamination in collagen and elastin. Although, the ECM modulation has been regarded as the main role of LOX, it also participates in regulation of migration, adhesion, and gene transcription [259–261]. It has been reported that there were miR-30a down regulations in ATC samples which was correlated with aggressive TC and increased mortality. It also inhibited ATC cell migration and invasion via LOX suppression [115]. Transgelin-2 (TAGLN2) is an actin-bundling protein involved in regulation of cell migration. There was significant miR-613 down regulation in PTC tissues that was correlated with lymph node metastasis. MiR-613 inhibited PTC cell invasion and EMT via TAGLN2 targeting [116]. Glutathione peroxidase 4 (GPX4) is a phospholipid hydroperoxidase that protects membranes toward the lipid peroxidation. CircKIF4A promoted the PTC cell migration while inhibited ferroptosis by miR-1231 sponging and subsequent GPX4 up regulation [117].

### MiRNAs involved in apoptosis and protein degradation

XIAP belongs to the family of apoptosis inhibitor proteins. It has been reported that the miR-618 over expression repressed ATC cell migration and invasion by targeting XIAP [118]. B-cell lymphoma 2 (BCL-2) belongs to the BCL-2 family that either inhibits or induces apoptosis. It has been shown that there was significant miR-15 up regulation in TC cells. It enhanced the MDA-T35 apoptosis and was also correlated with BCL-2 suppression. Migration assay confirmed the inhibitory role of miR-15 on MDA-T35 cells migration [119]. TP73 belongs to the p53 protein family that is involved in cell cycle and apoptosis regulation. CASC7 suppressed the PTC cell proliferation and migration by miR-34a-5p sponging and TP73 up regulation [120].

PSMD10 is one of the components of 26 S proteasome that negatively regulates tumor progression [262]. It has been reported that there was a significant reduced levels of miR-214 in PTC tissues and cells. It also suppressed PTC cell migration and EMT via PSMD10 targeting. Moreover, miR-214 down regulation was associated with lymph node involvement, tumor size, and TNM stage. Down regulation of PSMD10 also suppressed the Akt/GSK-3β/b-catenin cascade in PTC cells [121]. SMURF1 functions as an ubiquitin ligase that participates in viral autophagy, embryogenesis, and bone homeostasis [263–265]. It has been reported that there were miR-4319 down regulations in TC tissues and cell lines which were correlated with tumor size, lymphatic metastasis, and TNM clinical stage. The miR-4319 also repressed the TC cell migration and EMT through SMURF1 targeting [122]. SH3RF3 is an ubiquitin ligase involved in regulation of JNK pathway. There was miR-192-5p down regulation in PTC tissues and cell lines. MiR-192-5p reduced PTC cell migration and EMT, while increased apoptosis by SH3RF3 targeting [123].

## Conclusions

Despite recent progresses in TC diagnosis and treatment, there is still a high rate of tumor relapse and metastasis in these patients. Since, the early stage tumor detection can significantly reduce the tumor cell migration and relapse; it is necessary to suggest novel tumor markers for the early detection. MiRNAs as the critical regulators of tumor cell migration and progression, have higher stability in body fluids compared with mRNAs. Therefore, miRNAs can be suggested as non-invasive markers to predict the tumor relapse in TC patients. MiRNA-based treatment can be performed by targeting the deregulated miRNAs via anti-miRNA oligonucleotides and molecular sponges. However, the side effects can be observed because of the negative impact of anti-miRs on normal physiological functions. Therefore, site-specific delivery of anti-miRs by the effective methods is an important issue to reduce optimal concentration and side effects. Vectors and nano-carriers are the main delivery methods for the anti-miRs that can be effective in combination with chemotherapy drugs in TC treatment.

Taking miRNAs from bench to clinic requires safe delivery methods, restriction of off-target effects, and reduced toxicity and immune responses. The development of miRNA based treatments consists of the preclinical studies and clinical trials. Expression analysis of specific miRNA is quantified through PCR-based methods. MiRNAs are validated via reporter gene assays vectors, miRNA mimics, or inhibitors. Design of therapeutic miRNAs requires regulating the expression of candidate miRNAs through stabilization and encapsulation into nano-carriers and characterization for specific targeting. In vitro studies are also required to evaluate the selectivity, affinity, toxicity, dose-response, and the effects of miRNA-loaded nano-carriers on multiple biological processes in cell lines. Animal studies for candidate therapeutic miRNAs are also needed to evaluate the toxicity and biosafety, animal behavior, and pharmacokinetics. The clinical trial studies require a powerful evaluation of single and multiple doses, side effects, and long-term follow-ups in pre-clinical studies.

In present review, we have summarized all of the miRNAs that have been involved in thyroid tumor cells migration and invasion to pave the way of introducing a non-invasive diagnostic and prognostic panel of miRNAs in TC. We categorized the reported miRNAs based on their involved molecular processes to clarify their roles during thyroid tumor cells migration. It was observed that the miRNAs mainly exert their roles in thyroid tumor cell migration and invasion through regulation of PI3K/AKT and WNT signaling pathways. However, whether miRNA based treatment can be effective in suppression of TC tumor relapse and migration, needs further animal studies to approve the long-term safety of delivery tools in clinical practice. Moreover, the majority of clinical studies about the role of miRNAs in thyroid tumor cell migration have been based on the expressional analysis in tumor tissues. Indeed, it is also necessary to evaluate the circulating levels of miRNAs in TC patients to suggest them as non-invasive diagnostic markers.

## Data Availability

The datasets used and/or analyzed during the current study are available from the corresponding author on reasonable request.

## Abbreviations

TC: Thyroid cancer
WDTC: Well-differentiated TC
PDTC: Poorly-differentiated TC
ATC: Anaplastic thyroid carcinomas
PTC: Papillary thyroid carcinoma
FTC: Follicular thyroid carcinoma
MI-FTC: Minimally invasive FTC
WI-FTC: Widely invasive FTC
EMT: Epithelial-to-mesenchymal transition
E-CAD: E-cadherin
PI3K: Phosphatidylinositol-3-kinase
FOXN3: Forkhead Box N3
YAP1: Yes-associated protein 1
ceRNA: Competitively endogenous RNA
JAK: Janus kinases
ROCK: Rho-associated kinase family
SPHKs: Sphingosine kinases
PKC-θ: Protein kinase C-theta
PAK1: P21-activated kinase
HMGA2: High- mobility group-A2 gene
ncRNAs: Non-coding RNAs
lncRNAs: Long non-coding RNAs
miRNAs: MicroRNAs
circRNAs: Circular RNAs
YY1: Yin Yang 1
PcG: Polycomb-group
BCL-2: B-cell lymphoma 2
RCA: Rolling circle amplification
SDA: Strand displacement amplification
LAMP: Loop-mediated isothermal amplification
TAGLN2: Transgelin-2
GPX4: Glutathione peroxidase 4
